# The Case of Sulphuretted Hydrogen Gas Poisoning

**Published:** 1851-07

**Authors:** Theodore S. Bell


Art. III.—*The Case of Sulphuretted Hydrogen Gas poisoning*, by Theodore S. Bell, M. D.,

In the May number of this Journal I reported a case of poisoning by Sulphuretted Hydrogen gas, and endeavored to give as faithful a picture of the case under review, as a sincere desire to be faithful and accurate would enable me to give. The report of that case has proved to be the groundwork of one of the most scurrilous and vituperative assaults it has ever been my lot to read. Every principle of professional decency and courtesy was thrown aside in the article referred to; misrepresentation and error reigned supreme, the most absurd and ridiculous medical statements and opinions were uttered with as much gravity as though they had been tested by long experience, and were among the foundation stones of practical medicine. It is a matter of some importance that a portion of this extraordinary exhibition of Medical courtesy and liberality shall be examined and fairly tested, in order that we may find out what is the truth, and to whom its utterance belongs. The truth of my report shall be thoroughly established.

Before entering, however, upon this examination, I must express my obligations to Dr. Henry M. Bullitt, the author of the precious morceau in question, for the opportunity he has given me for doing justice to the long cherished regard I have felt for Dr. Powell, since the commencement of a friendship of many years duration.

There is no gentleman in the profession for whom I feel more regard, nor one for whose medical opinions I entertain more respect. A professional intercourse with Dr. Powell, of many years existence, has proved one of the most pleasant of my medical career, and entertaining such feelings as these, it would be a singular thing for me to select Dr. Powell as the object of public insult, and grossly improper treatment. The act would have been without a motive, and could have plead no excuse. A recent number of the Transylvania Journal has made a charge of this kind of conduct, the text of a singularly ludicrous homily on medical ethics, in which a violation of the practical part of all medical courtesy, largely predominates over the sermonizing. The accusation is based upon the remarks, in the report alluded to, which I made upon the use of water as an agent for the destruction of sulphuretted hydrogen gas. At the time those remarks were made, so far from having any knowledge that the use of water was in obedience to professional advice, I had strong reasons for believing that the water was used *against* medical advice. The only history I had of the matter, on the night of the disaster, was from Dr. C. L. Lyle, one of the witnesses paraded before the readers of the Transylvania Journal. Dr. Lyle stated to me that he was present when the water was used, and that he opposed its use while the victims were in the pit; that he entreated the people to stop its use, but, he said, they seemed to be wild, and poured the water in torrents. This was all the reliable testimony before me when my criticism was made, and I rested upon its faith. That testimony said very plainly that science opposed a popular fallacy, and I knew no better, until Dr. Powell himself informed me of his connection with the use of the water. Of Dr. Fry's agency I never heard until I saw it announced in the Transylvania Journal. I owe this explanation to Drs. Powell and Fry, and assure them that had I known of their connection with the order for the use of the water, my critisismwould have been of a different character. I should have contented myself with the expression of a preference for the use of fire rather than water. One of the last things I should have done, would have been to commit a wanton, unprovoked and motiveless attack upon the reputation of two gentlemen who possess a large share of my esteem and regard.

I should have preferred dropping this part of the subject with this *amende* to Drs. Powell and Fry, but Dr Bullitt's course forbids the exercise of such a preference. Without undertaking to affirm or deny my former remarks on the use of water in the destruction of sulphuretted hydrogen gas, I shall examine Dr. Bullitt's chemistry, physiology, toxicology, and therapeutics, for the Dr. has contrived to make quite a lively display on all these points. My erudite volunteer instructor informs my deplorable dullness with the refreshing knowledge that water absorbs sulphuretted hydrogen gas, a fact which I have a vague recollection of having heard before. But Dr. Bullitt instructs me only half way, for he fails to inform me that water parts with sulphuretted hydrogen gas quite as readily as it absorbs it. By way of reciprocity, I would inform Dr. Bullitt that water can be poured into a seive, but my impression is that it will not tarry long in that utensil. If Dr. Bullitt had earnestly pressed his surprising and laborious investigations, he would have found that Sir M. Brunel was much annoyed by sulphuretted hydrogen gas in the construction of the Thames tunnel. The workmen sickened, and a number of them died. The subject was referred to an accomplished toxicoligist, and upon examination he found that the gas escaped from the action of the river on iron pyrites, and the large volume of the river Thames was not able to absorb and hold the gas. It continued to annoy the workmen for months, shooting out upon them at unexpected times. Now with such a well known fact as this before my mind, am I to believe Dr. Bullitt's gratuitous assertions, and unsustained speculations? I differ from the high authority of Dr. Bullitt, with great deference, but even Aristotle was not infallible. Dr. Bullitt should have sustained himself with authorities, and closed the question upon me, and he will no doubt do it—when he can find them. Instead of taunting me with my ignorance of the "authorities" it would have been commendable in Dr. Bullitt to have shown some acquaintance with them himself. If he can find one "authority" in toxicology who gives any kind of countenance to his views of the "physiology of respiration;" if he will produce one who even leans towards an approbation of his notion that the best thing to be done for a patient, who is immersed in a gas that has already "partially or entirely suspended respiration," is to force him to respire this deadly gas again, I will surrender at discretion. Now let the erudite Dr. Bullitt wade among the tomes. I have made him a fair proposition, and will abide by its terms. There is no escape for Dr. Bullitt—he must either find "authorities" for his notions of exciting persons to the breathing of sulphuretted hydrogen gas, or he must admit that his indecent references to my ignorance were sadly out of joint as coming from him.

Dr. Bullitt says that it is entirely proper, when patients are in an atmosphere of gas "so irrespirable as to either partially or entirely suspend respiration," to force them to breathe it again. If so, why be in a hurry to get the patients out of the gas? why not play on the "physiology of respiration" with water, until the patients recover? Dr. Bullitt would have us believe that in proportion to the baleful effects of the gas, must be the effort of the physician to make the patient breathe more. According to Dr. Bullitt, in a case where respiration is only partially suspended, the patient may be safely left to gasp at the delightful gas, but when the poison has entirely suspended respiration, efforts must be made to arouse the patient to renewed struggles for more of the gas! For my part, probably for want of what my "general knowledge did not supply me," I would not desire a healthy man to breathe sulphuretted hydrogen gas, and I would not try to force a man to do it, who had already got a sufficient quantity in him to suspend respiration. Dr. Bullitt may consider me very stupid in not being convinced by his intellectual processes, but I cannot help it—I will not believe that any human being should be made to breathe sulphuretted hydrogen gas. I did all I could to submit my mind to the guidance of Dr. Bullitt, because I know the exceeding value of his medical opinions.

But let us proceed:

Dr. Bullitt says: "these men" (those in the pit) "were not in an atmosphere of absolutely irrespirable gas, but by the inhalation of sulphuretted hydrogen, respiration had become partially or entirely suspended, in consequence of the impression of the poison upon the nervous centers." This is Dr. Bullitt's reasoning, not Dr. Powell's nor Dr. Fry's. It is with Dr. Bullitt's presentation of the argument that I am now concerned, and as we are in the hands of a great master of physiology, beggars may pick up nutritive crumbs from his well stored table. I think myself very fortunate in receiving gratuitous instruction from Dr. Bullitt in chemistry and physiology, and the reader will find the extent of my obligations when I get through with this investigation.

Dr. Bullitt asserts that "respiration was suspended in consequence of the impression of the poison upon the nervous centers." Such famous reasoners as Dr. Bullitt are not expected to prove anything, but I should like to know what proof there is for the above reckless assertion. The statement, however, shows how ignorant Dr. Bullitt is of the subject on which he attempted to write. The attention of Dr. Bullitt was directed to the "physiology of respiration," and in the whole of the assault upon me, the entire coterie were so taken up with the respiratory movements, that there is not a hint thrown in all their learned writings that sulphuretted hydrogen gas can be fatal in any other way than by the lungs. This one fact shows how little any of the writing champions knew of what they were writing about. They expect the profession to believe them when they say that patients immersed in sulphuretted hydrogen gas, are properly treated if the respiratory functions can be forced to act. *But sulphuretted hydrogen gas is just as fatal to human life when acting upon the skin, as when breathed*, a fact stated by all the principal toxicologists. If a man were immersed to his neck in sulphuretted hydrogen gas, and his breathing organs were protected so as to have nothing to breathe but atmospheric air, the gas would as certainly kill him, as though he breathed it. What then becomes of all of Dr. Bullitt's twaddle about nervous centers, physiology of respiration, &c.? I feel exceedingly happy in the fact that I am not a professor of general physiology, for the profession might expect me to understand some of the *particulars* of my department. They would at least expect me not to introduce Hahneman's *similia similibus curantur* into a pit filled with sulphuretted hydrogen gas, as regular scientific treatment, as Dr. Bullitt does, when *he* proposes to cure persons in whom respiration is entirely suspended by sulphuretted hydrogen gas, by making the victims breathe more of the gas.

Such raw material may do to hash up for very green students, but no intelligent mind will ever swallow it with any expectation that assimilation will take place. The man who has to feed largely on such stuff is to be pitied.

On Dr. Bullitt's physiological principles, there was no necessity for any haste in getting the victims out of the pit, for water would rouse them to breathing, and that was enough. But I am informed that Drs. Powell and Fry made the most commendable efforts to remove the patients as soon as possible, and to that activity the victims are mainly indebted for their lives. Dr. Bullitt has such confused notions of the subject, that he endorses the statement that Mr. Atkinson was saved by the presence of mind that enabled him *not* to breathe the gas, and declares that the victims in the same pit, where Mr. Atkinson saved his life by not breathing, were saved by being made to breathe a gas that was so little irrespirable as to be able only to suspend respiration entirely. And this is my gratuitous, volunteer instructor, who dispenses knowledge that I can safely and prosperously dispense with!

But I am not done with my volunteer instructor. After Dr. Bullitt's sneer at my non-consultation of authorities, our readers may be interested in seeing a specimen of Dr. Bullitt's understanding of authorities. The Doctor taunts me with my ignorance, and in order to prove that it is proper to make people breathe irrespirable gas by pouring water upon them, quotes the following from Orfila: "*Exposure of the patient to the open air, aspersions* with cold vinegar and water, friction with a strong hair brush. These form the first assistance to be given to persons afflicted by privies." I cannot see what assistance Dr. Bullitt gets from this statement of Orfila's judgment. It condemns Dr. Bullitt's entire views of the treatment, and sustains the opinions advanced in my report. The very first thing is to get *the patient into the open air*, *away from the gas*, and then cold water or vinegar is to be *sprinkled* over the sufferers. Is this what Dr. Bullitt advocates when he talks about making the patients respire the noxious gas, by *affusions* of cold water? If I could make no better use of Orfila than this, I should let him rest in quiet.

Once more on the "authorities!" In my former notice I intended to show that fire was a better means for ridding a pit of sulphuretted hydrogen gas, than water, and Dr. Bullitt grows indignant on the subject, complains of my ignorance, and makes an admirable display of his own.—

With what kind of countenance can Dr. Bullitt look at the following quotation, from the most recent writer on medical jurisprudence, one of the best and highest of the authorities? Taylor says: "The best plan for getting rid of the gas is by a free exposure of the locality, *or by exciting active combustion in it.*" The latter recommendation could easily have been accomplished without injuring the persons in the pit. Dr. Bullitt must hunt up his authorities, and he must find them, or occupy a very unenviable position. I give him the run of Guy, Apjohn, Christison, Parent Duchatelet, Taylor and Michael Lèvy, and if he can find anything in them to sustain him, I yield every thing. But he must remember that I never objected to the *aspersion* of cold water on persons poisoned by the sulphuretted hydrogen gas, *after they were removed to a pure atmosphere.* If Dr. Bullitt were to see a drowning man, gasping for atmospheric air, under water, he would keep the man in the water because that fluid rouses "the physiology of respiration," and make the gasping man breathe more water, that fluid not being absolutely irrespirable. This would not be my course, and this constitutes the difference between Dr. Bullitt and myself. But the Doctor is a Professor of Physiology and I am not, and that may make a species of compensation or balance.

If Dr. Bullitt has really discovered a new method of treating patients poisoned with sulphuretted hydrogen gas; if he stands in direct opposition to every authority known in toxicology, and is so deeply imbued with fresh knowledge on the "physiology of respiration," as to be able to assert that the proper method of treating patients almost deprived of life by breathing a fatal gas is to make them breathe more of the noxious agent; if he is well established in the facts that the respiratory functions alone are to be attended to, and that the toxicologists are wrong in declaring that the action of the gas is just as fatal when applied to the skin, as when it is breathed: if

Dr. Bullitt is firm in all these novelties, he owes it to the profession to establish the doctrine. A field of glory and honor is before him; when he accomplishes his mission, he will be hailed as one of the great benefactors of his species, and the flood-tide of fame awaits him. Even I, humble as my merits are, will join in weaving a chaplet of laurel for his honored brow, and I may at least gather the glory of having appreciated his merits.

It would be reasonable to suppose that after such a spreading of all the glories of intellection, as I have recorded, Dr. Bullitt would have shown some signs of exhaustion, but he has marvellous gifts of continuance. He endorses the following extraordinary statement:

"We were much gratified in a few minutes by observing that he" (Mr. Ferguson) "began to breathe freer, his pulse at the wrist could be felt, and it seemed to get stronger and stronger, after each inspiration; at the expiration of fifteen or twenty minutes, Dr. Lyle, Fry and myself considered Mr. Ferguson in a fair way of recovery; his breathing was now full and regular, but slightly stertorous, his pulse both as to volume and frequency perfectly natural; there still remained, however, a profound coma; our only apprehension was the effects of the venous congestion of the brain, and we therefore determined to bleed him from the arm."

It is difficult to characterise this statement in terms that are at once suitable and becoming, but there is scarcely the shadow of truth in it. The attempt to connect Dr. Lyle with the statement is atrocious. That gentleman was an active, faithful and efficient coadjutor with Dr. Lewis Rogers and myself in the treatment of the case referred to, and the object in trying to use his name in the above connection is too obvious to require comment. Dr. Lyle never authorized his name to be used as confirmatory of the above statement, and he went over the items of it with me, and declared that the breathing was not full and regular, and the pulse was not natural, both as to volume and frequency. At the time when these symptoms were said to be present, I was sitting by the side of the patient; there were long intervals when there was no attempt at breathing—all the attempts that were made were spasmodic gaspings after air—my finger was on the wrist almost constantly for five hours, and at no time in all that period did the pulse make any approach to naturalness. During the time the patient laid in the porch, the pulse was often absent, and when present, could scarcely be felt. Just before the removal of the patient from the porch, Dr. Fry asked me respecting the pulse, and after feeling it some time, I replied that it was scarcely perceptible. He manifested no kind of surprise in finding that it had lost its "natural volume and frequency," but on the contrary, looked quite like himself.

I here re-publish my true statement of the condition of the patient in the porch: "The entire surface of the body was cold and apparently dead—the breathing, if the spasmodic attempts after air that were made by the sufferer can properly be called breathing, seemed to carry no air to the lungs. Mustard had been applied, but it produced no effect, and ammonia to the nose gave no sign ot its presence. It would be difficult to exaggerate the gravity of the symptoms. The skin was universally cold, as cold as death itself could make it; the spasmodic attempts to get air, already described; a pulse sometimes manifesting itself in a feeble flutter, and at other times altogether absent, and this state of things persisted in fully for three hours, and to a considerable extent for six hours, constituted one of the most serious forms of imminent danger I have ever seen."

Now it would be difficult to find a much greater difference between two statements, than is found between the one endorsed by Dr. Bullitt and the one I gave. I think that my word is entitled to quite as much credibility as

Dr. Bullitt's, and that far we are even. Dr. Bullitt attempts to lean on Dr. Lyle, in making his endorsement, but, Dr. Lyle says, without any authority from him. Dr. Lyle read over with me, since Dr. Bullitt's publication, each item of my description, and affirms that each and every part of it is true, and that the symptoms I described were such as were present. The witness thus relied upon by Dr. Bullitt sustains every statement I made as to the symptoms in the case. He coincides with me in the declaration that the symptoms thus recorded, remained in full existence for three hours, consequently, they were present when Dr. Rogers came into the case, an hour after I took part in it, and Dr. Rogers sustains my statement in every particular. Dr. Lyle says they were present from the time I reached the case to the time that Dr. Rogers joined us, and Dr. Rogers and Dr. Lyle vouch for the full truth of the report. Dr. Rogers read the report carefully before it was published, and remarked to me that so far as the description of the case and the treatment were concerned, the report was as accurate as it could be. Dr. Lyle has heard the present written statements, and affirms they are correct. Certainly Dr. Lyle, Dr. Rogers and myself are able to know the condition of a patient, and there was the most full and complete unity of views among us in this case throughout our connection with it. I am willing to rest the matter on the corroborative testimony of Drs. Rogers and Lyle.

What then becomes of the paltry attempt to falsify the statement of my report, and to injure the character of three physicians who were honestly and faithfully performing their professional duties? The witness relied on deserts the conspirators, and is true to his own honorable instincts and to professional justice. And when it is needed, I can multiply proof in support of my description of the case.

But let us return to the physiological endorser. I have sounded the shallows of his chemical and physiological knowledge, and shall now attend to his character as a pathologist. While Dr. Bullitt was endorsing the statements I have quoted, I think he might have inquired into the consistency and reasonableness of his bond. The statement on the face of it is that "the patient was in a fair way of recovery," when it is admitted there was "a profound coma" and "stertorous breathing." What wonderful gifts of prognosis some men have! Here in this case, even in the condition described by the endorser, the prescience that could say that a patient with "profound coma, stertorous breathing," and threatened with venous congestion of the brain" was "in a fair way to recover," is beyond not only my gifts, but those of medical authorities. I should be humiliated in finding myself so far below the gifts of the great practitioner who endorses the decision, if it were not for the good company I am in. The decision that the patient was "in a fair way to recover" was made in a few minutes after the victim was drawn from the pit, but Dr. Lyle and myself, with eight hours close watching of the case, and Dr. Lewis Rogers with seven hours observation, were not able to say, at the end even of that period, that this same patient was in "a fair way to recover." Throughout the hours in which the three physicians watched together, neither of us was able to bring himself to the positive conclusion that the case would recover, and when we parted at 2 o'clock, eight hours after the disaster, neither of us three was free from the apprehension of a fatal termination, though the patient slept "sweetly." And it is upon such judgments as I have described, that Dr. Rogers, Dr. Lyle and myself are tried and condemned. But I submit this part of the case just as it stands, to the judgment of the profession. Dr. Rogers is widely known as one of the first practitioners in Kentucky, and Dr. Lyle is one of the most promising of the young physicians in the city, and throughout the treatment of the case in question, there was neither clashing nor diversity of views among us. These gentlemen confirm the full truth of the picture I drew of the case in which they were concerned with me. And medical men can easily understand the declaration that we who attended the case, understood it quite as well as those who did not see it while we were treating it. There are few practitioners of medicine who have not met with just such quackish machinations and trickery as have been meted out to the physicians of this case; small cunning, mean inuendo, falsehood and injustice, and physicians everywhere can understand the meaning of these things. The blow was especially levelled at me, but each attempt upon me in relation to the treatment must equally affect Dr. Rogers and Dr. Lyle. That Dr. Bullitt made *deliberate* misrepresentation in this case, I have no kind of doubt. He speaks of the perversions of other men as though he were the paragon of honor, truth and justice. Of his claims to the title we shall now make an estimate. In entering upon his noble and elevating course of conduct, a comparison of the treatment of the two cases, Dr. Bullitt says: "in the other case, the treatment of which was conducted by Dr. Bell." Here is a striking specimen of Dr. Bullitt's truthfulness. He knew that the treatment of the case was conducted by Dr. Lyle, Dr. Lewis Rogers and myself, because, independently of any other source of information, the fact was prominently set forth in my report, and Dr. Bullitt professes to have read it. But Dr. Bullitt wished to use Dr. Lyle as a witness against me, and pretends to be friendly to Dr. Rogers, and in this state of case, he unscrupulously made the statement which I have quoted. This specimen of Dr. Bullitt's truthfulness will serve to show how much reliance may be placed on the rest of his statements.

Again, Dr. Bullitt asserts that there were marked differences in the results of the cases. I exceedingly dislike to touch anything of this kind, for Dr. Fry has no friend who feels prouder of the just and honorable reputation he won in the management of the German's case, than I do. He showed himself worthy of a place in the most honorable of all professions by his manly bearing, and his judicious course. And I feel sure that Dr. Fry is too correct in his gentlemanly deportment and professional knowledge, to set up a claim, that a difference of results, in cases treated by himself on the one hand, and on the other by Drs. Rogers and Lyle, must of necessity be caused by differences of treatment. Dr. Fry is physician enough to know that there are a great variety of circumstances that may make a difference in the result of cases, independently of any differences of treatment. In knowing this, Dr. Fry seems to have the advantage of Professor Bullitt.

But let us look at Dr. Bullitt's points of comparison of the treatment of the two cases:

1st. "There was a marked difference in regard to the recovery of the two patients."

This is true, but in a sense widely different from Dr. Bullitt's meaning. The patient of Dr. Rogers, Dr. Lyle and myself was walking all over the city attending to his business, for many days before the German could leave his room.

2nd. In the case of the German, "where depletion was resorted to as soon as the breathing and the circulation were re-established, and by other appropriate treatment, the coma and convulsions were soon overcome, and the patient was restored by the succeeding morning."

Well, in our case, as soon as Dr. Rogers, Dr. Lyle, and myself found our patient in a condition for bleeding, we bled him, and we were better judges of when the state of things came on that required the lancet than those who were not in attendance on the case. Dr. Adair Crawford covers the distressed Professor Bullitt precisely in his remarks on bloodletting in coma. Dr. Crawford says:

"'where a person is suddenly deprived of sense and motion, the practitioner is expected by the alarmed friends to employ instantly some active and efficient means of relief, and it is a very general rule with the profession to resort to the lancet, should there even be any doubt in the mind of the practitioner respecting the propriety of such a course, he may still be driven to adopt it, in deference to the opinions of the public, who ignorantly conclude that because bloodletting affords relief in some sudden fits, it is equally essential in all. If the patient recovers the cure is attributed to the venesection; and if he dies, it is hastily inferred that nothing could have saved him. If we turn, however, to a calm consideration of the various and very opposite causes of comatose affections, it will appear a most obvious truth that nothing can be more contrary to the legitimate deductions of science, and more dangerous in practice than this indiscriminate and empirical use of the lancet. It has been seen that among the causes of coma, some are connected with morbid conditions of the substance of the brain, for the removal of which bloodletting is the only, if not a sovereign remedy; but that on the other hand coma is frequently to be ascribed to a deficiency or prostration of the nervous energy of the brain in which case it is evident that the substraction of any large quantity of the vital fluid can only have the effect of preventing the powers of the constitution from rallying, and of thus turning the scale against the patient's recovery. We have little doubt that some of the recoveries from coma after bloodletting ought to be considered as truly escapes from death, rather than cures."

The character of Dr. Bullitt's claims is well drawn by Dr. Crawford. I should place but little value upon Dr. Bullitt's opinion even if he had been present in the treatment of either case, but as he was snoring in bed, while Dr. Rogers, Dr. Lyle and myself were passing the night with our patient, endeavoring to save his life, Dr. Bullitt's opinion may be placed, where it belongs—at zero.

But to return. Dr. Bullitt would have his readers understand that the sufferings of our patient were protracted beyond those of the German. This shows either ignorance or recklessness, on the part of my great critic. Drs. Rogers and Lyle were invited by Dr. Fry at 10 o'clock on the night of the disaster, to walk across the street to see the German—when they returned, they thought our patient was in a better condition than the other. Various citizens were passing from one patient to the other up to a late hour of the night, and they uniformly described the relative conditions of the patients. Dr. Fry was over to see our patient after midnight, and Drs. Rogers and Lyle went over to see the German after that period, and during this time, being many hours after the German was bled, no claim was set up to any decided differences in the condition of the two cases. The relative position of the cases was freely spoken of among the attending physicians up to sometime after midnight, and no one claimed that the German was in a better condition than our patient. The comparisons made were uniformly in favor of our patient. For the truth of this statement I appeal to Drs. Rogers and Lyle. Dr. Lyle has had this read to him, and fully corroborates my statement. "The bleeding was delayed some hours," says our great practitioner. This is one truth, but Drs. Rogers and Lyle say it was not delayed beyond the period when it could be beneficial to the patient, and the benefit in our case was marked and decided at once.

3rd Dr. Bullitt says, " But in consequence of the delay of the bleeding, our patient continued to suffer for thirty six hours or more from coma, and was not really restored for several days." But according to Drs. Rogers, Lyle and Fry, representatives of the two cases, our patient was doing, at least as well up to 12 o'clock as the German, and no one of Dr. Bullitt's striking results of the difference in the time of bleeding was discoverable during the many hours that intervened between the time of bleeding the German, and the time we left our patient to repose on what we had done. In looking at Dr. Bullitt's extraordinary statements, I wonder that he did not inform his readers that our patient died for want of bleeding, while his protogee was able to walk home at 10 o'clock, and let his physicians go off to the enjoyment of a dance. These assertions would have been scarcely a greater remove from the truth than the statements Dr. Bullitt actually makes. But what are the facts? Dr. Bullitt says our patient was comatose for thirty six hours, in consequence of the delay of the bleeding. But in my former report of the case I stated that our patient spake within ten hours of his attack, a fact well known to his family. There was not a symptom of coma in the case at that time, nor afterwards, as may be seen by referring to my report in the May No. of this Journal, page 413, a report made at a time when I had no reason to suppose that any human being would fabricate such calumnies as those made by Dr. Bullitt. Instead of the existence of coma, for thirty six hours, our patient walked down stairs, at his daughter's house, to a carriage, rode down home, got out of the carriage, walked up his lawn, then walked up stairs to his lounge, and walked up and down stairs as much as he pleased through the day, and all this without assistance, and this commenced within Dr. Bullitt's thirty six hours of coma!

Drs. Rogers and Lyle, know that at our early visit next morning, within fourteen hours after the disaster, there was not a trace of coma, and we ceased using remedies. Dr. Rogers, Dr. Lyle, and myself were actively performing our professional duties while Dr. Bullitt was dreaming all the things with which he has adorned the pages of a Medical Journal. What a frightful nightmare the great physiologist must have had on that eventful night.

Dr. Bullitt says our patient "was not really restored for several days." Now nothing is more notorious in this city than the fact that our patient was walking all over the city attending to his large business, a number of days before the German was able to leave his room. Two weeks after the accident, Mr. Charles Taylor informed me that the German was still confined to his room, while our patient was confined but a single day! Nor is the German now, on the 25th of June, more than two months after the disaster, able to attempt to work, as Mr. Taylor, his employer, informed me he had ascertained by calling on him on the date I have given. So much for the marked results of the difference of treatment in the two cases! "Our friends" may well exclaim, save us from Dr. Bullitt's pen.

It would be hard to conceive of a more unscrupulous and reckless course than that which has marked Dr. Bullitt's officious intermeddling with this affair, and I do not envy him the position he has won by his folly and malice. What particular interest of science he has advanced, what great boon he has conferred on the healing art, what improvement he has made in the minds of his readers, by his conduct must be left to time to determine. In private practice, a physician who slanders a brother practitoner respecting the treatment of a case, who is reckless in his statements, and calumniates the best and purest conduct of his professional brethren, is justly regarded by all honorable physicians as degraded and infamous. Whether similar conduct on the part of the editor of a Medical Journal is not entitled to at least an equal measure of scorn and contempt, is left to the judgment of the profession. From such exemplars of professional courtesy, honor, decency and truth, as the author of the conduct I have described in this notice, I wish to be free during the remainder of my life.

I must add here that Dr. C. L. Lyle, authorises me to use his name as corroborative testimony for the various statements I have made respecting the case we treated together.

